# Visual Field Prediction using Recurrent Neural Network

**DOI:** 10.1038/s41598-019-44852-6

**Published:** 2019-06-10

**Authors:** Keunheung Park, Jinmi Kim, Jiwoong Lee

**Affiliations:** 1 Department of Ophthalmology, Pusan National University College of Medicine, Busan, Korea; 2 Department of Biostatistics, Clinical Trial Center, Biomedical Research Institute, Pusan National University Hospital, Busan, Korea; 3 Biomedical Research Institute, Pusan National University Hospital, Busan, Korea

**Keywords:** Vision disorders, Translational research

## Abstract

Artificial intelligence capabilities have, recently, greatly improved. In the past few years, one of the deep learning algorithms, the recurrent neural network (RNN), has shown an outstanding ability in sequence labeling and prediction tasks for sequential data. We built a reliable visual field prediction algorithm using RNN and evaluated its performance in comparison with the conventional pointwise ordinary linear regression (OLR) method. A total of 1,408 eyes were used as a training dataset and another dataset, comprising 281 eyes, was used as a test dataset. Five consecutive visual field tests were provided to the constructed RNN as input and a 6^th^ visual field test was compared with the output of the RNN. The performance of the RNN was compared with that of OLR by predicting the 6^th^ visual field in the test dataset. The overall prediction performance of RNN was significantly better than OLR. The pointwise prediction error of the RNN was significantly smaller than that of the OLR in most areas known to be vulnerable to glaucomatous damage. The RNN was also more robust and reliable regarding worsening in the visual field examination. In clinical practice, the RNN model can therefore assist in decision-making for further treatment of glaucoma.

## Introduction

Glaucoma is a leading cause of blindness worldwide^1,2^. It is a chronic, irreversible optic neuropathy characterized by the progressive loss of retinal ganglion cells (RGCs) and their axons. Structural changes in ganglion cells eventually result in functional impairment of the visual field^2^ and greatly impact quality of life. In practice, monitoring visual field examination and determining its progression is an important process in the prevention of vision loss.

However, proper interpretation of visual field progression is difficult. In particular, the visual field test contains a large number of random errors and fluctuations that result in a low signal-to-noise ratio. The fluctuations are more severe in glaucoma patients than in normal subjects^3,4^. The pattern of visual field progression over time substantially differs among patients^5,6^. Previous studies have attempted to predict visual field: McNaught *et al*.^7^ compared curve-fitting models and reported that the linear regression model was best for generating the most accurate predictions of future visual field status^8^. However, more recent studies have reported that models of increasing complexity result in superior predictions. Caprioli *et al*.^9^ compared linear, quadratic, and exponential models; they reported that exponential models resulted in the best fit. Murata *et al*.^10^ used a type of machine learning algorithm, variational Bayes linear regression (VBLR); they reported that it demonstrated superior performance, compared with pointwise linear regression.

Recently, with tremendous advancements in computer performance, artificial intelligence capabilities have also greatly improved. Vast computational capacity and improved neural network algorithms have enabled artificial neural networks with increasingly greater depth. Eventually, “deep learning algorithms” emerged, with performance nearly comparable to that of humans. The greatest advantage of machine learning is that it does not require a precise mechanism to resolve complex problems; rather, it learns such mechanisms independently. In many cases, there is not a complete theoretical understanding of the problem. Visual field progression is a prototypical complicated problem with many unpredictable errors and large variations among patients.

In the past few years, 1 deep learning algorithm, the recurrent neural network (RNN), has shown outstanding achievement. Along with the convolutional neural network (CNN), which is successful with respect to image recognition, RNN has demonstrated great success in sequence labeling and prediction tasks for sequential data. A well-known application of RNN is represented by Apple’s Siri and by Google Voice^11,12^. Language is a notable example of sequential data by nature; the RNN has shown good performance in processing of the natural language problem^13,14^. Unlike other neural networks, RNN maintains the history of input data within the neural network^15^; thus, RNN output is produced with consideration for past input. A series of visual field examinations also comprises sequential input, by which the RNN can better interpret the true progression of the visual field and more accurately predict the future, compared with conventional methods.

In this study, we built an RNN architecture that receives a series of visual field examinations and predicts future visual field damage. We then evaluated the performance of the RNN by comparison with the conventional ordinary linear regression method (OLR).

## Methods

This was a retrospective study, performed in accordance with the tenets of the Declaration of Helsinki. The study was approved by the institutional review board (IRB) of Pusan National University Hospital; the requirement for patient consent was waived by the IRB because of the retrospective nature of the study.

All training and test data were obtained from subjects who had visited the glaucoma clinic at Pusan National University Hospital (South Korea) from 2005 to 2018. Subjects who had minimum of 6 consecutive visual field examinations were included in both training and test datasets. The training dataset consisted of 1408 eyes of 841 subjects; its demographic characteristics are summarized in Table 1. Training data were not labeled by diagnosis. Therefore, normal visual field data, as well as data from subjects with glaucoma and other optic neuropathies, were included; retinal disease and ocular media opacity (such as cataract) could also affect the visual field data. Subjects’ mean follow-up duration (years) and age were 4.5 ± 1.8 and 58.9 ± 16.2 (mean ± SD), respectively. The average initial visual field mean deviation (MD) was −7.02 ± 6.09 (mean ± SD). A total of 1408 records from the training dataset was randomly split into training data + validation data at a ratio of 9:1. Validation data were used to check the current fitness of the neural network during training to prevent overfitting.

**Table 1:** Demographic characteristics of the training dataset.

Demographics	Value
Total number of patients	841
Follow up duration (years), mean ± SD	4.5 ± 1.8
Age (years), mean ± SD	58.9 ± 16.2
Initial visual field MD (dB), mean ± SD	−7.02 ± 6.09
**Number of eyes**	
- Total	1408
- visual field: MD ≥ –6 dB	803
- visual field: –6 dB > MD ≥ –12 dB	330
- visual field: –12 dB > MD	275

MD = visual field mean deviation; SD = standard deviation.

Apart from the training dataset, another dataset, 281 eyes from 281 subjects, was prepared as the test dataset. There was no patient overlap between training and test datasets. For all subjects in the test group, retrospective review was performed of the detailed results of ophthalmic examinations; these ophthalmic examinations included the following measurements: best corrected visual acuity (BCVA), slit-lamp examination, funduscopy, biometry using the IOL Master (Carl Zeiss Meditec, Dublin, CA, USA), central corneal thickness (CCT) using ultrasonic pachymetry (Pachmate; DGH Technology, Exton, PA, USA), and keratometry using Auto Kerato-Refractometer (ARK-510A; NIDEK, Hiroshi, Japan). Glaucomatous optic neuropathy was defined upon meeting 1 or more of the following criteria: focal or diffuse neuroretinal rim thinning, localized notching, cup-to-disc ratio asymmetry ≥0.2, and the presence of retinal nerve fiber layer defects congruent with visual field defects^16^. Normal subjects were defined as those with no history of ocular disease, intraocular pressure (IOP) < 21 mmHg, absence of glaucomatous optic disc appearance, and a normal visual field.

### Visual field examination

Automated perimetry was performed by using a Humphrey Visual Field Analyzer 750i instrument (Carl Zeiss Meditec) with the Swedish interactive threshold algorithm (SITA) 24-2 or 30-2. Among 54 test points of the 24-2 test pattern, 2 points of physiologic scotoma were excluded; the remaining 52 test points were used. The 30-2 test pattern was converted to 24-2 by using overlapped test points. Reliable visual field tests were defined as false-positive rate <33%, false-negative rate <33%, and fixation loss <33%. Normal subjects were defined as those with a glaucoma hemifield test (GHT) within the normal limits, and with mean deviation (MD) and pattern standard deviation (PSD) within 95% of the normal population. Glaucomatous visual fields were those that met at least 1 of the following criteria: GHT outside the normal limits and/or PSD probability outside of 95% of the normal population.

### Artificial neural network

The open source neural network platform, Keras library, running on the tensorflow^TM^ (Google, Mountain View, CA, USA) python API r1.10, was used. Python language version 3.5 was used with CUDA toolkit 9.0 and cuDNN 7.0 library to utilize GPU computation power. The hardware environment was Intel i5-8400 CPU, 32 GB RAM, and two Geforce 1080Ti video cards (NVIDIA, Santa Clara, CA, USA) connected with an SLI bridge.

**Figure 1:**
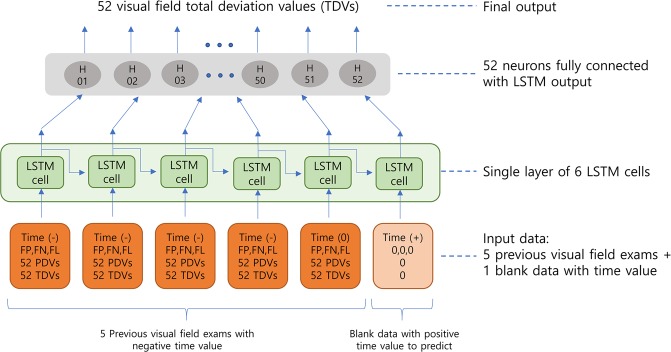
Recurrent neural network architecture. The total number of trainable parameters in the neural network architecture was 3,124 (2760 in LSTM layer + 364 in fully connected dense layer). Input data comprised 3 categories: relative time displacement in days, reliability data, and visual field data. Time displacement was defined as the most recent examination, and was set to zero; the past was indicated by using negative values and the future was indicated by using positive values (in days). Reliability data comprised false positive rate (FP), false negative rate (FN), and total fixation loss rate (FL). Visual field data comprised 52 pattern deviation values (PDV) and 52 total deviation values (TDV) of 24-2 Humphrey automated perimetry (2 points of physiologic scotoma were excluded). For normalization, total deviation values were divided by 50 before they were supplied to the neural network. The “input 0” is a special form of input data to provide the neural network with a future date for prediction. This input data contained only a positive time displacement value and all other values were set to zero. LSTM: long short-term memory, PDV: pattern deviation value, TDV: total deviation value, VF: visual field.

The final deep neural network architecture used in this study is shown in Fig. 1. A state-of-the-art RNN architecture, long short-term memory (LSTM), was used. A single layer of 6-LSTM cells received input data comprising 52 total deviation values (TDV), 52 pattern deviation values (PDV), reliability data (false negative rate, false positive rate, and total fixation loss rate), and time displacement value. Before they were fed into the neural network, TDV, PDV, and time displacement values were respectively divided by 50, 50, and 10000, for the purpose of normalization. Time displacement value was defined as the number of days from the most recent visual field examination. For example, the most recent visual field examination has the time displacement value of “0,” whereas the visual field examination that was performed 1 month (−31 days) prior to “0” has the time displacement value of “−31.” A negative sign in the time displacement value indicates that the examination was performed in the past.

Of the 6 consecutive visual field input data elements, 1 input data element contained a special format with positive time displacement (i.e., the point in the future that the user wishes to predict); all other data were set to 0. This special input was used to give the neural network information regarding the date that the user wishes to predict. A series of input data were arranged by reducing the time displacement value (i.e., from future to past) and then supplying this information to the neural network. If the total number of input data elements surpasses 6, which exceeds the input window of the neural network, such data can serve as the most recent 6 visual field examinations first; then, the next data can be supplied by sliding the time window 1 step into the future until the last input data are reached. However, in this study, we prepared all training and test datasets with exactly 6 consecutive visual field examinations, because we did not have a sufficient number of subjects with more than 6 visual field examinations. In the future, we plan to perform a multicenter study to collect sufficient data.

The LSTM layer is connected to the next single fully connected layer (dense layer), which consists of 52 neurons. These 52 neurons generate a final visual field prediction (1 neuron generates 1 visual field test point). This final RNN architecture was determined experimentally. We tested many different neural network architectures by varying the number of LSTM layers, the number of fully connected layers, the activation function, and the input data fed into the LSTM layer. The best neural network architecture was a single layer of LSTM with a single-layer fully connected network.

### Statistical analyses

To compare the performance of prediction, root mean square error (RMSE) and mean absolute error (MAE) of TDV were used as accuracy metrics. The same accuracy metrics were used in previous studies^10,17^. RMSE was calculated per each eye by using the equation below.

$$\begin{array}{rcl}RMSE & = & \sqrt{\sum _{n=1}^{52}\frac{{(trueTD{V}_{n}-predictedTD{V}_{n})}^{2}}{52}},\\ n & = & {n}^{th}\,test\,point\,of\,visual\,field\,exam\end{array}$$

MAE was calculated per each test point of the visual field throughout all eyes by using the equation below.

$$\begin{array}{lll}MA{E}_{n} & =\, & \sum _{i=1}^{number\,of\,eyes}\frac{|true\,TD{V}_{i,n}-predicted\,TD{V}_{i,n}|}{number\,of\,eyes}\\ n\, & = & {n}^{th}\,test\,point\,of\,visual\,field\,exam,\,i={i}^{th}\,eye,\\ TD{V}_{i,n} & = & total\,deviation\,value\,of\,{i}^{th}\,eye,\,{n}^{th}\,test\,point\end{array}$$

With those formulas, RMSE or MAE of RNN and OLR were calculated, respectively. Because accuracy metrics were paired (RNN, OLR), we used a pairwise test for comparisons. Depending on its normality, paired *t*-test or Wilcoxon’s signed-rank test were used to evaluate a significant difference in accuracy metrics (RMSE or MAE) between RNN and OLR. We performed Spearman’s correlation analysis as well as simple linear regression analysis to observe both parametric and nonparametric tests. They were used to investigate trends of prediction errors according to various factors such as false positive ratio, false negative ratio, and fixation loss. The Shapiro-Wilk test was performed to check the normality of the data distribution. In all statistical analyses, SPSS (version 21.0 for Windows; SPSS, Chicago, IL, USA) was used and a value of *P* < 0.05 was considered to indicate statistical significance.

## Results

A total of 281 eyes from 281 subjects were used for the test data set. The demographic characteristics are shown in Table 2. Mean follow-up duration was 5.1 ± 2.0 years and mean prediction time (the time interval between prediction and the last visual field examination) was 1.3 ± 0.3 years. The mean initial age of the patients was 63.2 ± 14.4 years. Initial visual field mean deviation (MD) was −6.35 ± 5.20 (dB).

**Table 2:** Demographic characteristics of the test dataset.

Demographics	Value
Follow up duration (years), mean ± SD	5.1 ± 2.0
Prediction time interval (years), mean ± SD	1.3 ± 0.3
Age (years), mean ± SD	63.2 ± 14.4
Sex (male/female), number	150/132
Spherical equivalence (Diopter), mean ± SD	−1.71 ± 3.40
Axial length (mm), mean ± SD	24.10 ± 1.71
Central corneal thickness (µm), mean ± SD	544.5 ± 35.1
**Visual field exam**	
- Initial MD (dB), mean ± SD	−6.35 ± 5.20
- Initial VFI (%), mean ± SD	88.0 ± 15.2
**Number of eyes**	
- Total	281
- Normal	30
- Glaucoma suspect	39
- Ocular hypertension	26
- Open angle glaucoma	73
- Normal tension glaucoma	79
- Angle closure glaucoma	15
- Pseudoexfoliation glaucoma	3
- Others	16

MD = visual field mean deviation; SD = standard deviation; VFI = Visual Field Index.

**Figure 2:**
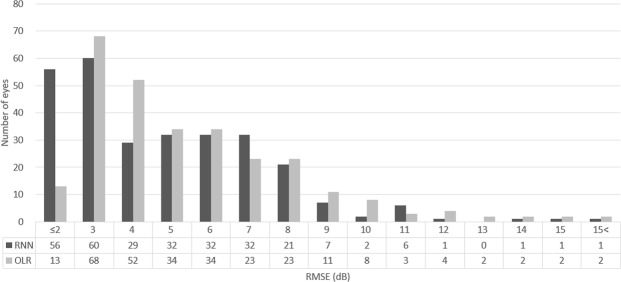
Number of eyes binned by prediction error (RMSE). The most frequent ranges of prediction error by RNN were ≤2 dB (56 eyes, 19.9%) and 2–3 dB (60 eyes, 21.5%); the corresponding ranges of prediction error by OLR were 2–3 dB (68 eyes, 24.2%) and 3–4 dB (52 eyes, 18.5%). OLR: ordinary linear regression, RMSE: root mean square error, RNN: recurrent neural network.

The number of eyes binned by RMSE prediction error is shown in Fig. 2. The most frequent ranges of prediction error by RNN were ≤2 dB (56 eyes, 19.9%) and 2–3 dB (60 eyes, 21.5%); the corresponding ranges of prediction error by OLR were 2–3 dB (68 eyes, 24.2%) and 3–4 dB (52 eyes, 18.5%). The largest difference was observed in the range ≤2 dB (56 vs. 13 eyes, RNN vs. OLR); above 4 dB, the frequency was similar between RNN and OLR.

**Figure 3:**
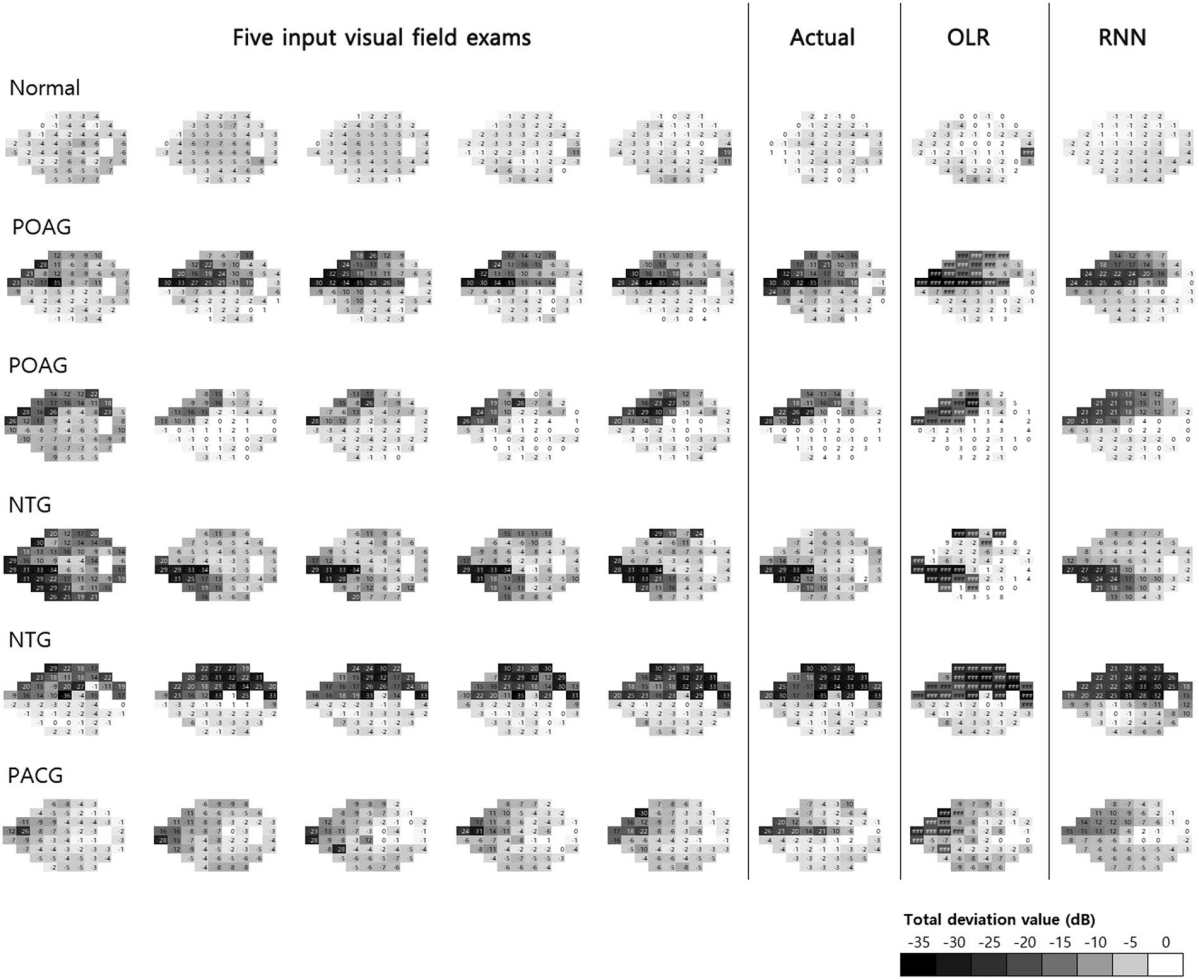
Representative examples of visual field predictions. Five consecutive input visual field examinations are shown in the left column followed by the actual visual field examination, predicted by OLR and RNN. In OLR, the outliers of past examinations are reflected, but RNN predictions were smoother. NTG: normal tension glaucoma, OLR: ordinary linear regression, PACG: primary angle closure glaucoma, POAG: primary open angle glaucoma, RNN: recurrent neural network.

Mean RMSE values of prediction according to disease are summarized in Table 3 and representative examples are shown in Fig. 3. In all subjects, the mean prediction error of the RNN (mean ± SD) was 4.31 ± 2.54 dB and that of OLR was 4.96 ± 2.76 dB; these were significantly different (*P* < 0.001). With the exceptions of angle-closure glaucoma and pseudoexfoliation glaucoma, the prediction RMSE of RNN was significantly better than OLR in all diseases. Notably, in the other diseases (optic neuropathy other than glaucoma), the RNN showed low prediction error, resulting in a larger difference between OLR and RNN (Δ_OLR−RNN_ = 1.53 dB). In angle-closure glaucoma alone, the RNN showed a larger prediction error (5.27 ± 2.52 dB) than that of OLR (5.09 ± 3.38 dB); however, this was not significant (*P* = 0.394).

**Table 3:** Comparison of mean RMSE between RNN and OLR.

	Prediction error (RMSE, dB), mean ± SD			*P* value
	RNN	OLR	Δ_OLR−RNN_	
All patients	4.31 ± 2.54	4.96 ± 2.76	0.65	<0.001^*^
- Normal	2.94 ± 2.13	3.57 ± 2.15	0.63	<0.001^*^
- Glaucoma suspect	3.40 ± 2.88	4.29 ± 3.13	0.89	0.003^*^
- Ocular hypertension	3.77 ± 3.33	4.43 ± 2.99	0.66	0.043^*^
- Open angle glaucoma	5.29 ± 2.00	5.81 ± 2.44	0.52	0.004^†^
- Normal tension glaucoma	4.62 ± 2.15	5.23 ± 2.46	0.61	0.008^*^
- Angle closure glaucoma	5.27 ± 2.52	5.09 ± 3.38	−0.18	0.394^†^
- Pseudoexfoliation glaucoma	3.95 ± 2.04	5.80 ± 0.85	1.85	0.285^†^
- Others	3.08 ± 2.80	4.61 ± 3.57	1.53	0.009^*^

OLR = ordinary linear regression; RMSE = root mean square error; RNN = recurrent neural network; SD = standard deviation.

^*^Paired t-test.

^†^Wilcoxon’s signed rank test.

**Figure 4:**
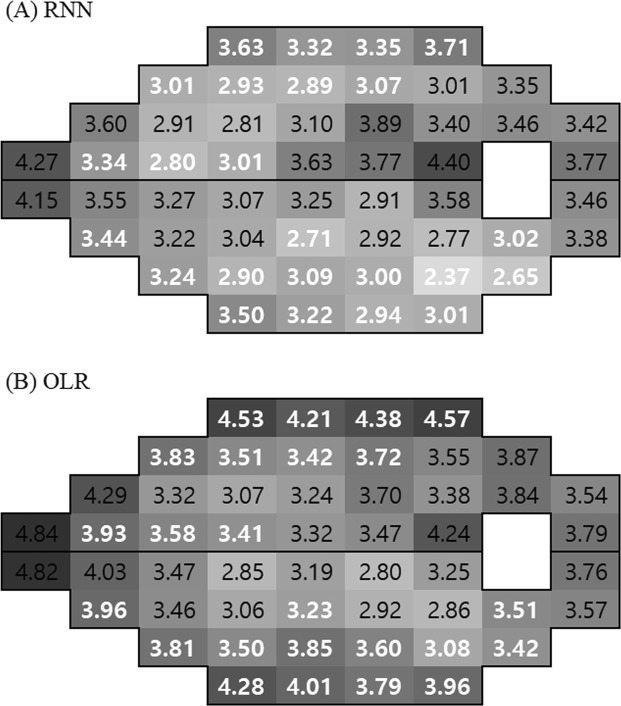
Pointwise mean absolute error (MAE) of predicted total deviation value (TDV). The darker color indicates higher error. White numbers indicate significant differences between RNN and OLR (paired t-test); black numbers are not significant. RNN showed significantly better performance, especially in superior, inferior, and temporal areas that are important in glaucomatous progression. OLR: ordinary linear regression, RNN: recurrent neural network.

Visual field test pointwise prediction error (MAE) is shown in Fig. 4. Of the 52 visual field test points, RNN showed a lower prediction error than OLR in 43 points; among these, 23 points were significantly different (shown in white numbers). Those significantly different points were generally located in superior, inferior, and temporal areas, which are typically vulnerable to glaucomatous damage. In 9 points, OLR was slightly better than RNN, but this difference was not significant, and was primarily located in the central area.

**Figure 5:**
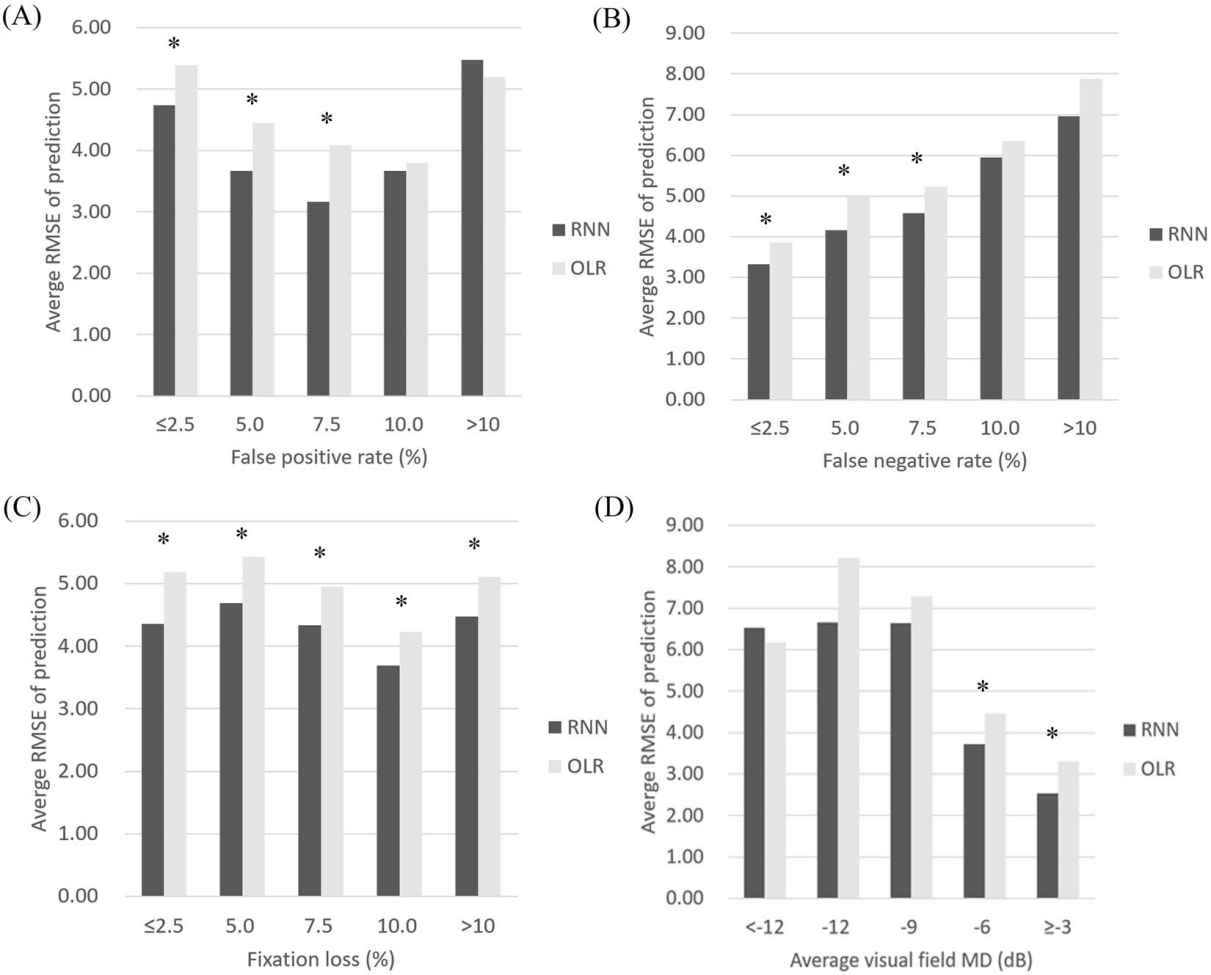
Average prediction error (RMSE) binned by various factors. (**A**) RMSE vs. false positive rate (**B**) RMSE vs. false negative rate (**C**) RMSE vs. fixation loss (**D**) RMSE vs. visual field mean deviation (MD). In general, RNN almost always showed lower prediction error than OLR. RMSE uniformly increased in both RNN and OLR as the false negative rate increased. False positive rate and visual field MD were considered to demonstrate a possible linear relationship with RMSE, but this trend was not uniform. RMSE vs. fixation loss was considered to demonstrate no obvious linear relationship. The symbol, asterisk (*), on top of the bar plot indicates the difference between RNN (dark gray bar) and OLR (light gray bar) is statistically significant. MD: mean deviation, OLR: ordinary linear regression, RMSE: root mean square error, RNN: recurrent neural network.

Mean RMSE values binned by various factors are summarized in Table 4 and Fig. 5. In RMSE vs. false positive rate, the prediction error of RNN was significantly lower than that of OLR until the false positive rate was <7.5. As the false positive rate increased, the difference between RNN and OLR became smaller and ultimately reversed, but was not significant (Table 4 and Fig. 5A). In RMSE vs. false negative rate, both RNN and OLR showed a prediction error that became greater as the false negative rate increased. However, RNN always showed lower prediction error than OLR; this was significant when the false negative rate was <7.5 (Table 4 and Fig. 5B). In RMSE vs. fixation loss rate, RNN always showed significantly lower prediction error than OLR (Table 4 and Fig. 5C). In RMSE vs. visual field MD, the prediction error of both RNN and OLR generally became greater as the visual field MD became worse; except for visual field MD <−12 dB, RNN showed lower prediction error than OLR. Notably, this difference was significant when MD was >−6 dB.

**Table 4:** Correlation coefficients and linear regression analyses between prediction error and reliability, and between prediction error and visual field MD.

	Correlation coefficients		Linear regression analysis			
	Spearman’s rho	*P* value	Slope	Intercept	r^2^	*P* value
**Prediction error vs false positive rate**						
RNN	−0.230	<0.001	−0.140	4.710	0.020	0.016
OLR	−0.226	<0.001	−0.181	5.483	0.029	0.004
**Prediction error vs false negative rate**						
RNN	0.442	<0.001	0.302	3.004	0.210	<0.001
OLR	0.452	<0.001	0.337	3.508	0.221	<0.001
**Prediction error vs fixation loss rate**						
RNN	−0.026	0.664	−0.001	4.317	<0.001	0.960
OLR	−0.043	0.469	−0.005	4.998	<0.001	0.884
**Prediction error vs average visual field mean deviation (MD)**						
RNN	−0.734	<0.001	−0.312	2.482	0.380	<0.001
OLR	−0.618	<0.001	−0.255	3.471	0.215	<0.001

OLR = ordinary linear regression; RMSE = root mean square error; RNN = recurrent neural network.

**Figure 6:**
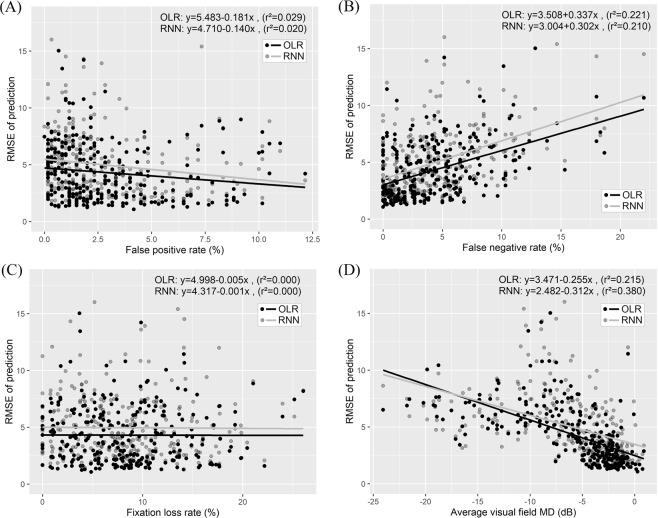
Linear regression analysis between prediction error (RMSE) and various factors. (**A**) RMSE vs. false positive rate (**B**) RMSE vs. false negative rate (**C**) RMSE vs. fixation loss (**D**) RMSE vs. visual field mean deviation (MD). Black circles and lines represent RNN and gray circles and lines represent OLR. False positive percentage demonstrated a significant relationship with prediction error; however, its r^2^ was low. False negative percentage showed a significant relationship with prediction error and a high r^2^ value. Fixation loss showed no significant relationship with prediction error. Visual field MD showed a significant relationship with prediction error and a high r^2^ value. MD: mean deviation, OLR: ordinary linear regression, RMSE: root mean square error, RNN: recurrent neural network.

The correlation coefficients and linear regression analyses between prediction error and various factors are shown in Table 5 and Fig. 6. The prediction error (RMSE) of RNN and OLR was significantly correlated with false positive rate, false negative rate, and visual field MD (all *P* < 0.001), but not with fixation loss rate (*P* = 0.664 vs. *P* = 0.469, RNN vs. OLR). Interestingly, both RNN and OLR had negative correlation with false positive rate; thus, as the false positive rate became greater, prediction error became smaller. However, the strength of correlation was weak (Spearman’s rho = −0.230 vs. −0.226, RNN vs. OLR); in linear regression analysis, r^2^ was also small (0.020 vs. 0.029, RNN vs. OLR). Prediction error had moderate positive correlation with false negative rate (Spearman’s rho = 0.442 vs. 0.452, RNN vs. OLR); in linear regression analysis, r^2^ was 0.210 vs. 0.221 (RNN vs. OLR). Both RNN and OLR showed strong negative correlation with visual field MD (Spearman’s rho = −0.734 vs. −0.618); in linear regression analysis, r^2^ was 0.380 vs. 0.215 (RNN vs. OLR). In summary, prediction error had a moderate to strong relationship with false negative rate and visual field MD, indicating that the prediction error became greater as the false negative rate or visual field MD became worse. However, the prediction error had no or weak correlation with fixation loss and false positive rate.

**Table 5:** Mean prediction error (RMSE) binned by reliability indices and visual field MD.

	Mean prediction error (RMSE), mean ± SD		Number of eyes	*P* value
	RNN	OLR		
**Prediction error vs false positive rate (FPR, %)**				
FPR ≤ 2.5	4.73 ± 2.68	5.39 ± 2.87	166	<0.001^*^
2.5 < FPR ≤ 5.0	3.67 ± 1.97	4.45 ± 2.42	68	<0.001^*^
5.0 < FPR ≤ 7.5	3.17 ± 2.14	4.09 ± 2.89	24	0.003^*^
7.5 < FPR ≤ 10.0	3.67 ± 2.83	3.80 ± 2.23	15	0.125^*^
FPR > 10	5.47 ± 2.02	5.20 ± 2.06	8	0.578^†^
**Prediction error vs false negative rate (FNR, %)**				
FNR ≤ 2.5	3.33 ± 2.16	3.86 ± 2.03	111	<0.001^*^
2.5 < FNR ≤ 5.0	4.17 ± 2.08	5.00 ± 2.70	72	<0.001^*^
5.0 < FNR ≤ 7.5	4.57 ± 2.32	5.23 ± 2.82	50	0.028^*^
7.5 < FNR ≤ 10.0	5.95 ± 2.43	6.34 ± 2.23	22	0.190^†^
FNR > 10	6.96 ± 3.08	7.88 ± 3.24	26	0.052^*^
**Prediction error vs fixation loss rate (FLR, %)**				
FLR ≤ 2.5	4.36 ± 2.14	5.18 ± 2.58	43	0.016^*^
2.5 < FLR ≤ 5.0	4.69 ± 3.13	5.42 ± 2.95	41	0.001^*^
5.0 < FLR ≤ 7.5	4.34 ± 2.34	4.95 ± 2.64	51	0.013^*^
7.5 < FLR ≤ 10.0	3.69 ± 2.52	4.22 ± 2.45	56	0.001^*^
FLR > 10	4.47 ± 2.52	5.11 ± 2.95	90	<0.001^*^
**Prediction error vs average visual field mean deviation (MD, dB)**				
MD < −12	6.53 ± 1.61	6.18 ± 1.69	37	0.145^†^
−12 ≥ MD > −9	6.66 ± 2.09	8.21 ± 3.17	20	0.670^*^
−9 ≥ MD > −6	6.64 ± 2.76	7.28 ± 3.39	43	0.980^*^
−6 ≥ MD > −3	3.72 ± 1.66	4.46 ± 1.97	77	<0.001^*^
MD ≥ −3	2.53 ± 1.44	3.31 ± 1.57	104	<0.001^*^

OLR: ordinary linear regression, RMSE: root mean squared error, RNN: recurrent neural network, SD: standard deviation.

^*^Wilcoxon’s signed rank test between mean prediction error of RNN and OLR.

^†^Paired t-test between mean prediction error of RNN and OLR.

## Discussion

The main objectives of this study were to build a state-of-the-art deep learning algorithm, RNN architecture, to predict visual field examination, and then to evaluate its accuracy in comparison with the conventional linear regression method. The performance of RNN was considerably better than that of OLR. Overall prediction error (RMSE) was 4.31 vs. 4.96 (RNN vs. OLR), which was significantly different (*P* < 0.001). In almost all diseases, including optic neuropathy other than glaucoma, RNN yielded superior predictions to OLR. RNN was also more robust to the worsening of visual field reliability. Prediction accuracy worsened as the false negative rate of the visual field increased in both RNN and OLR; however, the prediction error of RNN was lower than that of OLR. To our knowledge, this is the first report utilizing RNN architecture to predict visual field examination.

Recently, deep-learning architecture has been used in glaucoma. However, many of these studies are limited to classifying the visual field rather than being involved in any predictions. Aaoka *et al*.^18^ constructed a deep-learning architecture to discriminate preperimetric glaucoma from normal glaucoma. Its diagnostic performance was 92.6% (area under the receiver operating characteristic curve, AUROC) and they reported the performance was superior to all other machine-learning methods such as random forests, gradient boosting, support vector machine, and neural networks. Kucur *et al*.^19^ developed a convolutional neural network (CNN), a kind of deep-learning architecture, to discriminate early glaucoma from normal glaucoma. They used two visual field examinations as input data, OCTOPUS 101 perimeter and Humphrey visual field 24-1. The average precision score performance of CNN was 0.874, which was better than conventional visual field global indices, with a mean defect square root of the loss variance. However, unlike us, the cited authors used a neural network to discriminate glaucoma from normal eye status; they did not seek to predict the outcomes of future visual field examinations. Yousefi *et al*.^20^ compared the performance of various machine-learning algorithms to detect glaucoma progression. They used both the retinal nerve fiber layer (RNFL) measured by optical coherence tomography (OCT) and the visual field mean deviation (MD) and pattern standard deviation (PSD) as input data. The best performance was achieved by a random forest-tree algorithm with an AUROC of 0.88. However, the machine-learning algorithms used in those studies also did not predict future visual field test results.

There have been many efforts to precisely predict visual field; many have used mathematical regression models to fit a series of visual field examinations and predicted the next visual field by extrapolation. A pointwise linear regression model was simple but reliable for prediction of the visual field. Bengtsson *et al*.^21^ reported reliable prediction in most patients by using linear extrapolation based on 5 initial visual field tests. McNaught *et al*.^7^ reported a similar result: they compared polynomial models to predict the next visual field by using 5 previous visual field tests; they found that the linear model provided optimal forecast of pointwise glaucomatous visual field progression. Other long-term studies have also reported that fitting by linear regression yielded the best model in a majority of patients^22–24^. Caprioli *et al*.^9^ developed a pointwise exponential regression model and reported that it better characterized fast or slow progression rate with respect to visual field damage, compared with linear models. More complex models have been developed to consider variations in the rates of glaucomatous damage over time^25^. Chen *et al*.^26^ reported that the average RMSE values of visual field prediction were 2.925 for logistic functions and 3.056 for exponential functions. More recently, Otarola *et al*.^25^ reported that a pointwise sigmoid regression model showed a mean RMSE of 4.1, and that it better characterized both early and late stages of glaucoma. However, an opposite study reported that of all exponential, quadratic, or logistic models, none exhibited accuracy superior to that of linear regression^27^.

Thus far, there have been few studies regarding the use of machine learning to predict future visual field. Murata *et al*.^10^ used the VBLR method to predict pointwise TDV. They evaluated the performance of the VBLR by varying the number of input visual field data elements from 2 to 10. Their overall RMSE was 4.5 ± 2.4 dB when 5 input data elements were used. Our RNN model showed an overall RMSE of 4.31 ± 2.4 dB, slightly better than that of VBLR. Because the test datasets are not identical, it is difficult to conclude that our RNN model is necessarily better than the VBLR approach. However, the number of training data elements differs widely between RNN (1408 eyes) and VBRL (5049 eyes) models, while the performance of RNN remains comparable and may be superior. In future studies, we expect better performance if we train the RNN with additional data. Yousefi *et al*.^20^ trained a machine learning algorithm with 2085 eyes and concluded that it could detect visual field changes earlier than other methods; however, their method did not predict pointwise visual fields. Rather, it more closely resembled a classifier regarding whether visual field changes will progress. The introduction of a deep learning algorithm to predict visual field examination is more rarely reported. To our knowledge, there was a single study by Wen *et al*.^28^ utilizing Cascade-Net, a type of CNN architecture, to predict future Humphrey visual field (HVF). This study is not formally published yet (it is in preprint status). However, their deep learning network showed excellent performance to generate predictions for future HVF (total threshold values) up to 5.5 years, given a single HVF as input. The neural network was trained with approximately 32,443 consecutive 24-2 HVFs, and the overall RMSE was 3.47 dB. However, these are not published data; moreover, the use of single visual field data as input may not reflect true progression of the visual field. Chauhan *et al*.^29^ recommended that at least three visual field examinations scheduled over 2 years were required to reliably detect progression. Even though Cascade-Net showed a better performance than we report herein, we presume direct comparison may not be possible.

To build a deep neural network architecture, we used LSTM cells, a unique type of RNN algorithm, because it exhibits some advantages with respect to conventional RNN. LSTM was first introduced by Hochreiter & Schmidhuber in 1997^15^. In their study, conventional RNN failed to learn when the time lag was >5–10 discrete time steps between relevant input data and output; thus, conventional RNN disregarded its input data too rapidly, because it must quickly vanish or backpropagated errors will multiply^30,31^. The LSTM is not affected by this problem because it utilizes separate internal states of memory and stores input data into the neural network for an extended period. To accurately predict the visual field, it has been reported that a minimum of 5 visual field examinations are required^32^. In this regard, the LSTM algorithm is more appropriate for the prediction of visual field progression than conventional RNN. In the year 2000, Gers *et al*.^33^ added an “adaptive forget gate” to the LSTM; this modified LSTM was more robust to noisy input because the “forget gate” releases its internal memory when data retention is no longer necessary. By enabling the release of unnecessary data, the LSTM became more robust to noisy input, known as the noisy temporal order (NTO) problem. Our model uses this modified LSTM with “forget gate”; we observed that the RNN exhibited significantly lower prediction error than OLR, even when the reliability of the visual field was worsened. We presume this was because we provided the neural network with a reliability index, as well as visual field data, and because the LSTM may selectively use the input data.

Among the reliability indices, the greatest influence on visual field predictions in our study was the false negative rate. However, the correlation coefficient of the false positive rate was weak (Spearman’s rho = −0.230 vs. −0.226, RNN vs. OLR); r^2^ in the linear regression analysis was also small (0.020 vs. 0.029, RNN vs. OLR), indicating that the contribution of the false positive rate to the RMSE was <3% in both RNN and OLR. Interestingly, fixation loss did not affect prediction accuracy in either RNN or OLR models; our results were similar to those of a previous study. Ramulu *et al*.^34^ reviewed 10,000 visual fields from 1,538 eyes; in their study, fixation losses were not significantly associated with unexpectedly high or low sensitivity at any stages of visual field damage, while the false negative rate and false positive rate contributed to the increased uncertainty. Rao *et al*.^35^ also reported similar results; they evaluated the effect of reliability indices (false negative rate, false positive rate, and fixation loss) on visual field assessment. In their study, the false negative rate significantly influenced visual field assessment, while the false positive rate and fixation loss were not associated with visual field assessment. In our study, the false negative rate was the only index that truly affected visual field prediction among the reliability indices.

In this study, RNN provided more accurate predictions than OLR in the inferior and superior regions of the visual field. Garway *et al*.^36^ reported that these regions of the visual field can be mapped to the superotemporal, superonasal, inferotemporal, and inferonasal regions of the optic nerve head; these regions are closely associated with glaucomatous damage^37^. We presume this is because the RNN considers all visual field values, while pointwise linear regression solely considers specific points. Visual field areas vulnerable to glaucomatous damage are more likely to progress stochastically. The neural network may have learned this trend in spatial distribution of visual field progression throughout the training dataset.

There were several limitations in our study. First, we trained and tested only 5 consecutive visual field data elements as input, because we did not have a sufficient number of patients with >6 visual field tests (5 for input + 1 for prediction). However, many previous studies^7,21,38^ have also reported the same number of input visual field data elements, and we are planning a multicenter study to collect additional patient data. In future studies, we will evaluate a varying numbers of input data and the optimal number of LSTM cells in our RNN architecture can vary slightly. Second, all visual field data were acquired from a single center. Thus, our RNN model may not be widely applicable.

## Conclusion

We constructed a novel deep learning architecture by utilizing a state-of-art LSTM algorithm, a type of RNN. Our RNN model predicted future visual field significantly better than a conventional pointwise linear regression method. This RNN model was also more robust to reductions in the reliability of visual field input data. In clinical practice, the RNN model can assist in decision-making for further treatment of glaucoma.

## Data Availability

The datasets generated during and/or analysed during the current study are available from the corresponding author on reasonable request.
