# Author Correction: Visual Field Prediction using Recurrent Neural Network

**DOI:** 10.1038/s41598-019-49490-6

**Published:** 2019-09-06

**Authors:** Keunheung Park, Jinmi Kim, Jiwoong Lee

**Affiliations:** 1 Department of Ophthalmology, Pusan National University College of Medicine, Busan, Korea; 2 Department of Biostatistics, Clinical Trial Center, Biomedical Research Institute, Pusan National University Hospital, Busan, Korea; 3 Biomedical Research Institute, Pusan National University Hospital, Busan, Korea

Correction to: *Scientific Reports* 10.1038/s41598-019-44852-6, published online 10 June 2019

This Article contains typographical errors in the Acknowledgements section.

“This research was supported by the Bio & Medical Technology Development Program of the National Research Foundation (NRF) funded by the Korean government (MSIT) (No. NRF-2018M3A9E8066253).”

should read:

“This research was supported by the Bio & Medical Technology Development Program of the National Research Foundation (NRF) funded by the Korean government (MSIT) (No. NRF-2018M3A9E8066254).”

